# Effect of Carbazochrome Sodium Sulfonate in Addition to Tranexamic Acid in Bleeding Trauma Patients

**DOI:** 10.7759/cureus.22018

**Published:** 2022-02-08

**Authors:** Yuka Okazaki, Hiroaki Takada, Ichiro Okada, Eiju Hasegawa

**Affiliations:** 1 Department of Critical Care Medicine and Trauma, National Hospital Organization Disaster Medical Center, Tokyo, JPN

**Keywords:** hemorrhage, bleeding, transfusion, trauma, carbazochrome sodium sulfonate, tranexamic acid

## Abstract

Background: It is important to evaluate the effects of drugs considered to control hemorrhage. Tranexamic acid (TXA) has been shown to reduce the risk of death in bleeding trauma patients. Carbazochrome sodium sulfonate (CSS) is often used in combination with TXA; however, it is unknown whether CSS additionally improves the control of bleeding in trauma patients.

Methods: The aim of this study was to examine whether CSS reduces blood transfusion and death in addition to TXA by improving the control of bleeding. We retrospectively analyzed medical records of trauma patients from 2011 to 2019. We included patients aged ≥16 years, with significant hemorrhage, and who received TXA within eight hours from injury as per CRASH-2 (Clinical Randomisation of an Antifibrinolytic in Significant Haemorrhage) study. The primary outcome was the total amount of red blood cells (RBC), fresh frozen plasma (FFP), and platelet concentrate (PC) received within the first 24 hours from injury. Secondary outcomes were death in hospital within four weeks after injury, vascular occlusive events, and treatment.

Results: During this retrospective evaluation period, 5764 admissions with trauma were registered. A total of 326 cases met the selection criteria: 259 cases who received CSS in addition to TXA (CSS group; n=259) and 67 cases who received only TXA (no-CSS group; n=67). The mortality rate was 6% in the no-CSS group and 15.1% in the CSS group. There was no significant difference in mortality and vascular occlusive events between the two groups. We performed multiple regression analyses, with the amount of blood transfusion for each type as explanatory variables. The administration of CSS was an independent factor for the reduction of RBC transfusion (standard partial regression coefficient −0.1, 95% CI [−3.1 to −0.1], p=0.04), but not for transfusion of FFP or PC. We also performed multiple logistic regression analysis, with death as an explanatory variable. CSS was not an independent factor for any cause of death.

Conclusion: CSS decreased RBC transfusion in trauma patients, without increasing the risk of vascular occlusion. However, CSS did not decrease mortality. This study can contribute to managing bleeding with trauma, but further research aimed at clarifying the effect of CSS is needed.

## Introduction

Every year, more than a million people die worldwide as a result of road traffic injuries [1]. Uncontrolled hemorrhage after an injury is a cause of preventable death [2]. Despite the improved hemorrhage control, many patients still die [3]. For the most critically injured patients requiring emergent surgery, overall mortality has changed and remains close to 50% [4,5]. Thus, it is important to evaluate the effects of drugs considered to control hemorrhage.

Tranexamic acid (TXA) has been shown to reduce the risk of death in bleeding trauma patients in the CRASH-2 (Clinical Randomisation of an Antifibrinolytic in Significant Haemorrhage) trial [1]. TXA is a synthetic derivative of the amino acid lysine; it inhibits fibrinolysis by blocking the lysine binding sites on plasminogen [6]. Carbazochrome sodium sulfonate (CSS) is often used in combination with TXA in bleeding patients [7]. However, the mechanism of action of CSS is not fully clear. CSS is a capillary stabilizer and is used clinically for the treatment of hemorrhage due to the fragility of capillaries [8]. To our knowledge, there have been no studies reporting on the efficacy of CSS or examining whether CSS improves the control of bleeding in addition to TXA in trauma patients with bleeding. CSS may also reduce the perioperative inflammatory response in total knee arthroplasty [9]; however, the anti-inflammatory effect of CSS has not been well examined in trauma.

The aim of this study was to examine whether CSS reduces blood transfusion and the possibility of reducing mortality death in addition to TXA by improving the control of bleeding.

## Materials and methods

Study design

We retrospectively analyzed the medical records of trauma patients from January 1, 2011, to January 1, 2019. The study was approved by the local ethics committee (No. 2020-10) and performed in accordance with the ethical standards stated in the Declaration of Helsinki.

Setting

This study was a retrospective cohort study conducted at a single center. The hospital is a tertiary health care center with a total of 455 beds, of which 34 are in intensive care. It is also a referral center supporting a region with a population of approximately one million people.

Selection of participants

The selection criteria for our study were in line with those from the CRASH-2 protocol [6]. We included patients aged ≥16 years, with significant hemorrhage (systolic blood pressure (SBP) <90 mm Hg or heart rate (HR) >110 beats/min, or both) or who were considered to be at risk of significant hemorrhage, and who received TXA within eight hours from injury. We excluded patients with cardiopulmonary arrest on arrival (CPAOA).

Procedure

We administered 1 g of TXA by an intravenous infusion without following to all patients at high risk of bleeding within three hours from the transfer. The choice to use CSS was also left to the physician's discretion and there were no clear selection criteria for CSS administration. Each patient in the CSS group received 50 mg CSS by an intravenous infusion without following. TXA was manufactured by Teva Takeda Pharma Ltd., Nagoya, Japan. CSS was manufactured by Nichi-iko Pharmaceutical Co., Ltd., Toyama, Japan.

Data collection

The following data were collected from the medical records: age; sex; Abbreviated Injury Scale (AIS) of each part, Injury Severity Score (ISS); Revised Trauma Score (RTS); the probability of survival (Ps); vital signs, such as Glasgow coma scale (GCS), body temperature (BT), HR, SBP, and diastolic blood pressure (DBP), respectively), and respiratory rate (RR); laboratory data, such as base excess (BE), hemoglobin (Hb), platelet count (Plt), activated partial thromboplastin time (APTT), international normalized ratio of prothrombin time (PT-INR), fibrinogen, fibrin degradation product (FDP), D-dimer, C-reactive protein (CRP), lactate (Lac); history of medication use, such as antiplatelet drug and anticoagulant drug; time since injury; type of injury (blunt or penetrating). We calculated shock index (SI)-HR divided by SBP, as well as the Assessment of Blood Consumption (ABC) score and the Trauma Associated Severe Hemorrhage (TASH) score. The ABC score is based only on non-laboratory and non-weighted parameters: SBP, HR, and positive Focused Assessment with Sonography in Trauma (FAST) examination [10]. The TASH score uses seven independent variables: SBP, hemoglobin concentration, FAST results, the presence or absence of complex long bone and/or pelvic fractures, HR, base deficit, and sex [11].

Outcome measures

The primary outcome was the total amount of red blood cells (RBC), fresh frozen plasma (FFP), and platelet concentrate (PC) delivered within the first 24 hours from injury.

Secondary outcomes were death in the hospital within four weeks after injury, vascular occlusive events (myocardial infarction, stroke, pulmonary embolism (PE), and deep vein thrombosis (DVT)), surgical intervention (neurosurgery, thoracic, abdominal surgery, and surgery of pelvic and extremities), and transcatheter arterial embolization (TAE). The cause of death was described by the following categories: bleeding, vascular occlusion (myocardial infarction, stroke, and PE), multiorgan failure (MOF), head injury, and others.

Statistical analysis

Results were expressed as median and interquartile ranges (IQR) unless otherwise stated. Demographic factors and baseline characteristics were summarized using descriptive statistics. The groups were compared using the Mann-Whitney U test. Categorical variables were analyzed using Fisher’s exact probability test. Multivariate regression analysis was performed for the primary outcomes, adjusted by possible confounders for massive transfusion (fibrinogen, FDP, and SI) [12-14] and for age and ISS. Multiple logistic regression analysis was performed for death, adjusted by clinically plausible factors (GCS, SI, and type of injury). All statistical analyzes were performed with EZR (Saitama Medical Center, Jichi Medical University, Saitama, Japan) and R (The R Foundation for Statistical Computing, Vienna, Australia). All tests were two-sided, and a p-value of <0.05 was considered statistically significant.

## Results

Results

**Figure 1:**
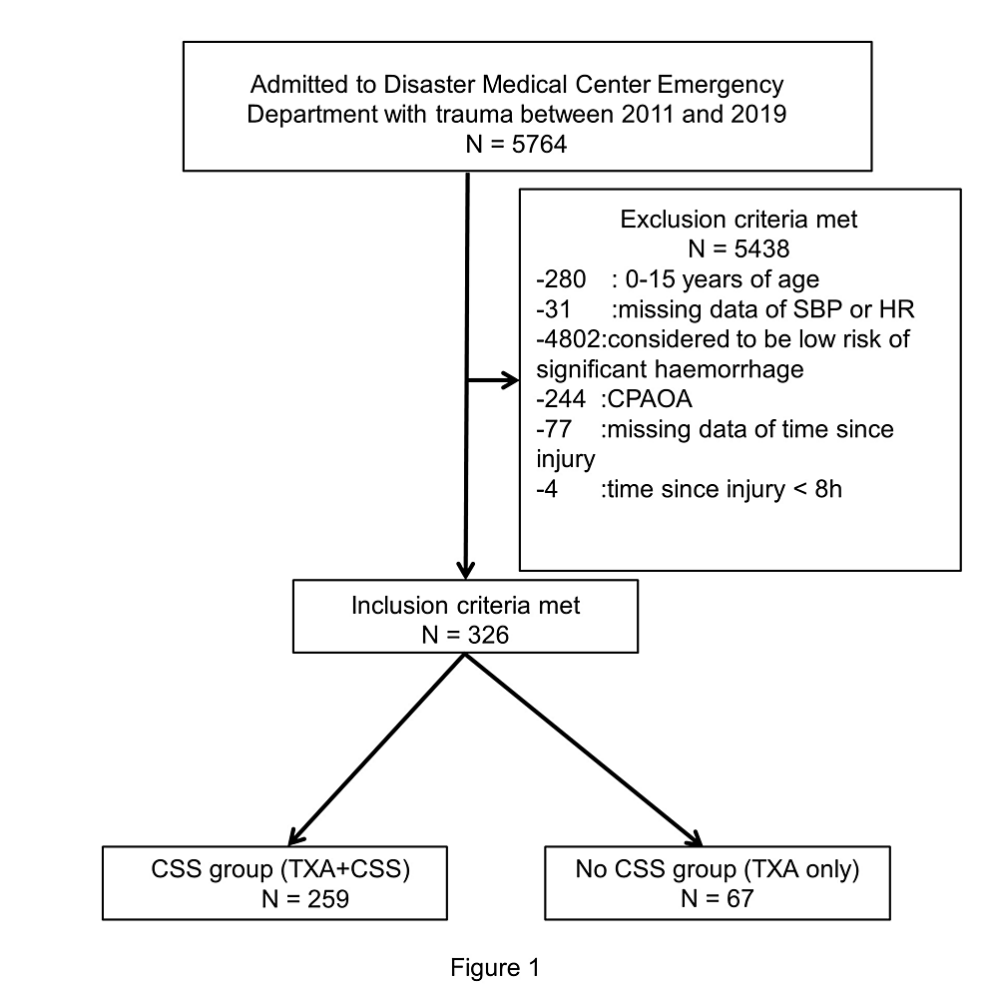
Flowchart of patient inclusion SBP: systolic blood pressure; HR: heart rate; CPAOA: cardiopulmonary arrest on arrival; CSS: carbazochrome sodium sulfonate; TXA: tranexamic acid.

During this retrospective evaluation period from 2011 to 2019, 5764 admissions with trauma were registered. A total of 280 cases were younger than 16 years, and 31 cases had no SBP or HR data. In addition, 4802 cases were considered not to be at high risk of bleeding, and 244 cases had CPAOA. The time since injury was unknown in 77 cases and was over eight hours in four cases. After the application of the exclusion criteria, 326 cases remained for analysis (Figure 1).

Baseline characteristics

A total of 259 cases in the CSS group received CSS in addition to TXA (CSS group; n = 259) and 67 cases were given only TXA (no-CSS group; n = 67). Patients’ baseline and clinical characteristics are summarized in Table 1. There were no significant intergroup differences in age, sex, time since injury, type of injury, history of medication, BT, HR, DBP, RR, Hb, Plt, FDP, fibrinogen, PT-INR, APTT, D-dimer, AIS score on the face, thorax, abdomen, pelvis and extremities, and surface, ISS, RTS, and CRP. The GCS score and AIS score on the head in the no-CSS group were significantly higher than those in the CSS group (p = 0.03). Lac was also higher than the CSS group (p = 0.02). On the other hand, SBP, and BE in the no-CSS group were significantly lower than those in the CSS group.

**Table 1:** Characteristics of trauma patients with bleeding * p < 0.05 BT: body temperature; GCS: Glasgow coma scale; HR: heart rate; SBP: systolic blood pressure; DBP: diastolic blood pressure; RR: respiratory rate; BE: base excess; Hb: hemoglobin; Plt: platelet count; FDP: fibrin degradation product; PT-INR: international normalized ratio of prothrombin time; APTT: activated partial thromboplastin time; AIS: Abbreviated Injury Scale; ABC: assessment of blood consumption; TASH: trauma-associated severe hemorrhage; ISS: Injury Severity Score; RTS: Revised Trauma Score; Ps: probability of survival.

Characteristic	All (n = 326)	No-CSS group (n = 67)	CSS group (n = 259)	p value
Age (years)	56.5 (33.2–74.0)	44 (30.5–71.5)	61 (35–74)	0.12
Sex (female), n (%)	100 (30.7)	17 (25.4)	83 (32.0)	1.00
Time since injury (h)	0.7 (0.6–0.9)	0.7 (0.6–1.0)	0.7 (0.5–0.8)	0.26
Blunt trauma, n (%)	282 (86.5)	55 (82.1)	227 (87.6)	0.23
Anticoagulant drug, n (%)	19 (5.8)	3 (4.5)	16 (6.2)	0.77
Antiplatelet drug, n (%)	17 (5.2)	3 (4.5)	14 (5.4)	1.00
BT (℃)	36.2 (35.6–36.6)	36 (35.6–36.6)	36.2 (35.6–36.7)	0.40
GCS score*	14 (9–15)	14 (11.3–15.0)	14 (8.3–15.0)	0.03
HR (bpm)	86 (73–101)	88 (74.5–100.0)	86 (72–102)	0.79
SBP (mm Hg)*	137 (117–156)	122 (109–152)	138 (119–158)	0.01
DBP (mm Hg)	82 (69.8–96.0)	79 (67.0–92.3)	83 (70–96.8)	0.10
RR (bpm)	20 (17–25)	21 (18–26)	20 (17–25)	0.39
BE (mmol/L)*	−1.4 (−4 to 0)	−3.4 (−5.0 to 0.3)	−1.1 (−3.5 to 0.1)	<0.01
Hb (g/dL)	13.1 (11.8–14.4)	12.5 (11.5–14.3)	13.1 (11.9–14.5)	0.14
Plt (×10^3^/μL)	21 (17.2–25.8)	20.5 (8.6–32.3)	21 (1.6–61.6)	0.75
FDP (μg/mL)	34.9 (0–117.3)	37.3 (10.7–158.1)	33.8 (0.0–111.0)	0.14
Fibrinogen (mg/dL)	218.5 (180.3–278)	207 (174.5–251)	222 (181–283.5)	0.23
PT-INR	1.1 (1.0–1.2)	1.1 (1.0–1.2)	1.1 (1.0–1.2)	0.09
APTT	27.1 (24–30.9)	25.7 (23.4–29.7)	27.4 (24.4–31)	0.06
D-dimer	22.5 (5.5–45.4)	22.7 (5.7–46.2)	20.1 (4.2–44.8)	0.58
AIS head*	3 (0–4)	3 (2–4)	0 (0–3)	<0.01
AIS face	0 (0–0)	0 (0–0)	0 (0–0)	0.66
AIS thorax	0 (0–3)	0 (0–3)	0 (0–3)	0.82
AIS abdomen	0 (0–2)	2 (2–3)	3 (2–3)	0.22
AIS pelvis + extremities	0 (0–2)	3 (2–3)	3 (2–3)	0.22
AIS surface	0 (0–1)	0 (0–1)	0 (0–0)	0.06
Shock Index*	0.6 (0.5–0.8)	0.7 (0.5–0.8)	0.6 (0.5–0.8)	0.03
ABC score	0 (0–1)	0 (0–1)	0 (0–1)	0.24
TASH score*	2 (1–5)	3 (2–7)	2 (1–4)	0.01
ISS	18 (9.3–26)	20 (13.3–26)	17 (9–29)	0.26
RTS	7.6 (6.0–7.8)	7.6 (6.0–7.8)	7.8 (6.9–7.8)	0.06
Ps*	0.94(0.77-0.98)	1.0 (0.9–1.0)	0.9 (0.7–1.0)	<0.01
CRP (mg/dL)	0.1 (0.1–0.2)	0.1 (0.1–0.2)	0.1 (0.0–26.2)	0.35
Lac (mmol/L)*	14 (5.3–22.8)	18 (10.1–32.0)	13 (5–19)	0.02

Outcomes and treatments

Table 2 shows the outcomes and treatments of trauma patients included in this study. The mortality rate was 6% (four cases) in the no-CSS group and 15.1% (39 cases) in the CSS group. There was no significant intergroup difference in mortality. The most common cause of death was head trauma in the CSS group. Death due to head trauma was significantly more common in the CSS group than in the no-CSS group. There was no significant difference between the two groups in the occurrence of other causes of death, including MOF and vascular occlusive events. As for treatments, significantly more head and abdominal surgeries were performed in the CSS group than in the no-CSS group. There were no significant intergroup differences in the proportion of thoracic surgery, surgery of pelvis and extremities, and TAE. The administration of RBC and FFP in the CSS group was significantly lower than that in the no-CSS group. The administration of PC was significantly lower in the no-CSS group than in the CSS group.

**Table 2:** Outcomes and treatments of trauma patients included in this study * p < 0.05 MOF: multiorgan failure; PE: pulmonary embolism; DVT: deep vein thrombosis; TAE: transcatheter arterial embolization; RBC: red blood cells; FFP: fresh frozen plasma; PC: platelet concentrate.

Outcome	All (n = 326)	No-CSS group (n = 67)	CSS group (n = 259)	p value
Any cause of death, n (%)	43 (13.2)	4 (6.0)	39 (15.1)	0.07
Bleeding, n (%)	1 (0.3)	0 (0)	1 (0.4)	1.00
Vascular occlusion, n (%)	2 (0.6)	1 (1.5)	1 (0.4)	0.37
Head injury, n (%)*	28 (8.6)	1 (1.5)	27 (10.4)	0.02
MOF, n (%)	1 (0.3)	0 (0)	1 (0.4)	1.00
Other causes, n (%)	11 (3.4)	2 (3.0)	9 (2.3)	1.00
Any vascular occlusive event, n (%)	9 (2.8)	3 (4.4)	6 (2.3)	0.40
Myocardial infarction, n (%)	1 (0.3)	1 (1.5)	0 (0.0)	0.21
Stroke, n (%)	5 (1.5)	1 (1.5)	4 (1.5)	1.00
PE, n (%)	2 (0.6)	1 (1.5)	1 (0.4)	0.37
DVT, n (%)	3 (0.9)	1 (1.5)	2 (0.8)	0.50
Any surgery, n (%)	160 (49.1)	36 (53.7)	124 (47.8)	0.41
Head surgery, n (%)*	51 (15.6)	5 (7.5)	46 (17.8)	0.04
Thoracic surgery, n (%)	4 (1.2)	1 (1.5)	3 (1.2)	1.00
Abdominal surgery, n (%)*	23 (7.1)	9 (13.4)	14 (5.4)	0.03
Surgery of pelvis+extremities, n (%)	84 (25.8)	21 (31.3)	63 (24.3)	0.27
TAE, n (%)	33 (10.1)	9 (13.4)	24 (9.3)	0.36
RBC (unit)*	0 (0–4)	0 (0–6)	0 (0–2)	<0.01
FFP (unit)*	0 (0–6)	2 (0–8)	0 (0–4)	<0.01
PC (unit)*	0 (0–0)	0 (0–40)	0 (0–50)	0.04

We performed multiple regression analyses, with the amount of blood transfusion for each type as explanatory variables (Tables 3-5). The administration of CSS in addition to TXA was an independent factor in the reduction of RBC transfusion (standard partial regression coefficient −0.1, 95% CI (−3.1 to −0.1), p = 0.04). However, the administration of CSS in addition to TXA was not an independent factor for transfusion of FFP or PC.

**Table 3:** Multiple regression analysis of various variables for the prediction of transfusion of RBC * p < 0.05 RBC: red blood cells; CSS: carbazochrome sodium sulfonate; FDP: fibrin degradation product; SI: shock index; ISS: Injury Severity Score.

	Standard partial regression coefficient	95% confidence interval	p value
CSS*	−0.1	−3.1 to −0.1	0.04
Fibrinogen*	−0.2	−0.0 to −0.0	<0.01
FDP	0.1	−0.0 to 0.0	0.28
SI*	0.3	3.1–7.3	<0.01
ISS*	0.2	0.0–0.2	<0.01
Age	−0.0	−0.5 to 5.4	0.50

**Table 4:** Multiple regression analysis of various variables for the prediction of transfusion of FFP * p < 0.05 FFP: fresh frozen plasma; CSS: carbazochrome sodium sulfonate; FDP: fibrin degradation product; SI: shock index; ISS: Injury Severity Score.

	Standard partial regression coefficient	95% confidence interval	p value
CSS	−0.1	−4.1 to 0.2	0.07
Fibrinogen*	−0.3	−0.0 to −0.0	<0.01
FDP	0.1	−0.0 to 0.0	0.23
SI*	0.2	2.8–8.7	<0.01
ISS*	0.2	0.1–0.2	<0.01
Age	−0.1	−0.1 to 0.0	0.37

**Table 5:** Multiple regression analysis of various variables for the prediction of transfusion of PC * p < 0.05 PC: platelet concentrate; CSS: carbazochrome sodium sulfonate; FDP: fibrin degradation product; SI: shock index; ISS: Injury Severity Score.

	Standard partial regression coefficient	95% confidence interval	p value
CSS	−0.1	−3.2 to 0.2	0.08
Fibrinogen*	−0.2	−0.0 to −0.0	<0.01
FDP	−0.0	−0.0 to 0.0	0.70
SI*	0.2	1.7–6.4	<0.01
ISS*	0.2	0.0–0.2	<0.01
Age	−0.0	−0.0 to 0.0	0.75

We also performed multiple logistic regression analysis, with death as an explanatory variable (Table 6). The administration of CSS in addition to TXA was not an independent factor for any cause of death.

**Table 6:** Logistic regression analysis of various variables for the prediction of death * p < 0.05 CSS: carbazochrome sodium sulfonate; GCS: Glasgow coma scale; SI: shock index.

	Adjusted odds ratio	95% confidence interval	p value
CSS	2.2	0.6–7.5	0.22
GCS*	0.7	0.6–0.8	<0.01
SI	0.7	0.2–2.2	0.55
Blunt	1.6	0.4–6.0	0.51

## Discussion

Our study in trauma patients revealed that CSS reduced the amount of RBC transfusion within the first 24 hours from injury compared with that in patients who only received TXA. However, CSS did not reduce mortality or the amount of transfusion of FFP or PC. CSS did not increase the risk of any vascular occlusion event. Significantly more head and fewer abdominal surgeries were performed in the CSS group than in the no-CSS group (Table 2).

CSS is widely used in trauma patients [7]. However, its mechanism of action is not yet clearly understood. Sendo et al. has reported that CSS reverses tryptase- and thrombin-induced endothelial barrier dysfunction, and inhibits bradykinin-induced hyperpermeability [8]. However, it has not yet been investigated whether there is an interaction between TXA and CSS hemostatic mechanisms. Therefore, the effects of co-administration of CSS and TXA are not clearly understood.

To the best of our knowledge, this is the first study to examine whether CSS can reduce the amount of RBC transfusion. Luo et al. performed a randomized controlled trial in patients undergoing total knee arthroplasty; it was shown that the amount of bleeding can be reduced by administering CSS in addition to TXA, without increasing complications [9], which is compatible with our results. However, the amount of blood transfusion was not evaluated in the previous study, presumably because blood transfusion is rarely required in TKA. In the case of trauma, it is difficult to measure the exact amount of blood loss. However, there should be a positive correlation between the amount of blood loss and the amount of blood transfusion. Even in TKA, which is considered to be less invasive than severe trauma, additional administration of CSS has been shown to decrease blood loss. This suggests that additional administration of CSS would also reduce blood loss and transfusion volume in severe trauma.

This is the first study to examine whether CSS can reduce death in trauma patients. In this study, CSS did not reduce mortality. However, although there was no significant difference, mortality tended to be higher in the CSS group. Similarly, RTS and Ps in the CSS group tended to be lower. This can be partly considered that CSS was administered more frequently to patients who were assessed as more severely injured by each physician based on vital signs and the first impression at the time of admission. Thus, we adjusted factors that make up the RTS score, such as the GCS score to reduce the bias when considering mortality in the two groups in multivariate analysis. In addition, TXA was proven to reduce death in bleeding trauma patients in the CRASH-2 study [1]. This previous study had a large sample size with 10,000 cases per group, while the sample size of our study was too small to show that CSS administration reduces mortality. Elimination of the bias and the larger number of cases would be needed to validate the reduced mortality of CSS in patients with severe trauma.

Our study suggests that CSS does not increase thromboembolism complications. In TKA, CSS combined with TXA did not increase the incidence of thromboembolic complications [9], which is comparable to our results. This may imply that CSS shows hemostatic effect without affecting blood coagulation and fibrinolysis.

In the present study, CRP and MOF mortality did not significantly differ between the two groups. Luo et al. indicated that CSS combined with TXA was more effective than TXA alone in reducing the perioperative inflammatory response including IL-6, erythrocyte sedimentation rate (ESR), and CRP [9]. Also, Hong et al. revealed the effects of reduction of gingival inflammation of fixed-dose combinations of vitamin C, vitamin E, lysozyme, and CSS in patients with chronic periodontitis [15]. The effect of CSS in these reports is inconsistent with our results. The development of organ dysfunction is related to the intensity and balance between trauma-induced simultaneous, opposite inflammatory responses [16]. Although there is an anti-inflammatory effect of CSS, the effect of CSS was maybe not clear due to too much tissue damage in trauma compared with TKA. Other inflammatory markers such as IL-6 or ESR and pain scores have not been examined in this study; they should be evaluated in detail if they are measured in the future.

Study limitations

This study has several limitations. First, it was a retrospective cohort study conducted at a single center, which introduces potential selection bias. Moreover, uncontrolled confounding factors may exist. However, it is difficult to randomize in the acute phase of severely traumatized patients. Second, no cases received CSS alone at our center, so we were not able to study the efficacy of CSS administered alone. Moreover, given that TXA is effective for bleeding trauma patients, it is ethically problematic to administer only CSS without TXA to trauma patients. Third, since the TXA administration method performed in this study is different from the CRASH-2 method, the results of this study may not be applicable if the TXA administration method is different. A few studies have examined the dose of TXA in trauma, and it is unclear what the problem is with the difference in dose from CRASH-2 in this study. Forth, the induction criteria for treatment are not unified. It was decided at the discretion of the attending physician to administer CSS or not. Fifth, the sample size was relatively small.

## Conclusions

In addition to TXA, CSS decreased RBC transfusion in trauma patients without increasing the risk of vascular occlusion events. However, CSS did not decrease death in bleeding trauma patients. This study can contribute to managing bleeding with trauma. Further research aimed at establishing the effect of CSS is needed.
